# NIA DIVISION OF NEUROSCIENCE

**DOI:** 10.1093/geroni/igae098.0333

**Published:** 2024-12-31

**Authors:** Molly Wagster, Eliezer Masliah

**Affiliations:** National Institute on Aging, Baltimore, Maryland, United States; National Institute on Aging, Baltimore, Maryland, United States

## Abstract

Dr. Masliah will discuss research priorities of the NIA Division of Neuroscience. Dr. Masliah will also be available for small group discussion.

